# *Interleukin-16* Polymorphism Is Associated with an Increased Risk of Ischemic Stroke

**DOI:** 10.1155/2013/564750

**Published:** 2013-10-31

**Authors:** Xiao-li Liu, Jian-zong Du, Yu-miao Zhou, Qin-fen Shu, Ya-guo Li

**Affiliations:** ^1^Department of Neurology, Zhejiang Hospital, Hangzhou, Zhejiang 310013, China; ^2^Department of Respiratory, Zhejiang Hospital, Hangzhou, Zhejiang 310013, China

## Abstract

Clinical and experimental data have demonstrated that inflammation plays fundamental roles in the pathogenesis of ischemic stroke. Interleukin-16 (IL-16) is identified as a proinflammatory cytokine that is a key element in the ischemic cascade after cerebral ischemia. We aimed to examine the relationship between the *IL-16* polymorphisms and the risk of ischemic stroke in a Chinese population. A total of 198 patients with ischemic stroke and 236 controls were genotyped using polymerase chain reaction-restriction fragment length polymorphism (PCR-RFLP) and DNA sequencing method. We found that the rs11556218TG genotype and G allele of *IL-16* were associated with significantly increased risks of ischemic stroke (TG versus TT, adjusted OR = 1.88; 95% CI, 1.15–3.07; G versus T, adjusted OR = 1.54; 95% CI, 1.05–2.27, resp.). However, there were no significant differences in the genotype and allele frequencies of *IL-16* rs4778889 T/C and rs4072111 C/T polymorphisms between the two groups, even after stratification analyses by age, gender, and the presence or absence of hypertension, diabetes mellitus, hypercholesterolemia, and hypertriglyceridemia. These findings indicate that the *IL-16* polymorphism may be related to the etiology of ischemic stroke in the Chinese population.

## 1. Introduction

Stroke is a complex multifactorial disorder and a leading cause of mortality and morbidity worldwide. Approximately, 70–80% of all cases are ischemic stroke (IS) [1]. Growing evidence has shown that inflammation appears to play crucial roles in the pathogenesis of IS through the promotion of atherosclerosis. Clinically, the incidence and outcome of IS are influenced by systemic inflammatory processes, and stroke patients with systemic inflammation portend poorer outcomes [2–4]. Experimentally, focal ischemic injury with acute and chronic inflammation induces production of proinflammatory mediators and recruitment of inflammatory cells, including neutrophils, T cells, and monocyte/macrophages [5–7]. In addition to some known risk factors, such as inflammation, advanced age, hypertension, and diabetes, genetic variants have been reported to contribute to the development of IS [8–10]. Identification of susceptibility genes is of great value to clarify the aetiology and improve prognostication, therapy, and prevention.

Interleukin-16 (IL-16) was originally discovered as lymphocyte chemoattractant factor in 1982 [11]. Subsequently, it was identified as a proinflammatory cytokine that is a 67 kD precursor protein consisting of 631 amino acids. The gene encoding IL-16 is located on chromosome 15q26.3 in humans [12–14]. IL-16 is produced by activated CD8-positive (CD8+) T cells [15] and activates CD4+ T cells, monocytes, macrophages, and dendritic cells by binding to the CD4 molecular [16, 17]. In addition to the activation of CD4+ T cells, IL-16 can promote the secretion of inflammatory cytokines such as tumour necrosis factor (TNF) *α*, interleukin-1*β*, and interleukin-6 (IL-6) [18]. All of these cytokines are key elements in the ischemic cascade after cerebral ischemia [19–22].

In 2000, Nakayama and colleagues found a -295T-to-C polymorphism in the promoter region of *IL-16, *which is related to different levels of gene expression [23]. The polymorphism was reported to be associated with a series of inflammatory or autoimmune diseases, including Crohn's disease [24], allergic contact dermatitis [25], endometriosis [26], asthma [27], Graves' disease [28], and systemic lupus erythematosus (SLE) [29]. Recently, another two functional polymorphisms (i.e., rs11556218 and rs4072111) were identified to be associated with SLE [29] and coronary artery disease (CAD) [30, 31]. To date, no data exist regarding the contribution of the *IL-16* polymorphisms to IS. In this study, we aimed to assess the prevalence of the *IL-16* polymorphisms in IS.

## 2. Materials and Methods

### 2.1. Study Population

The study was approved by the Zhejiang Hospital Institutional Review Board for Human Subjects Research, and written informed consent was obtained from all individuals. The study population comprised 434 unrelated Chinese subjects who visited the Zhejiang Hospital between March 2010 and September 2012. Among them, 198 were patients with IS (122 men and 76 women) and 236 were healthy controls (155 men and 81 women). IS was determined by the presence of a focal or global neurological deficit with symptoms and signs lasting more than 24 h. Computed tomography and/or magnetic resonance image scanning of the brain was used to ascertain the diagnosis of IS. The subgroups of IS were classified using the TOAST criteria [32]. Hypertension was defined as having a systolic blood pressure ≥140 mmHg and/or a diastolic blood pressure ≥90 mmHg. Diabetes mellitus was diagnosed if the fasting plasma glucose level ≥7.0 mmol/l or the plasma glucose ≥11.1 mmol/l 2 hours after a 75 g oral glucose tolerance test. Hypercholesterolemia was determined by a fasting serum cholesterol level >6.2 mmol/L, and hypertriglyceridemia was determined by a fasting serum triglyceride level >2.3 mmol/L [33]. We used hospital-based controls that were age, gender, and ethnicity matched, without a history of IS.

### 2.2. Genotyping

Genomic DNA was isolated from peripheral blood leukocytes by the salting-out method [34]. *IL-16* polymorphisms were genotyped by performing polymerase chain reaction-restriction fragment length polymorphism (PCR-RFLP) assay. Primer sequences and reaction conditions were used as described previously [35]. For quality control, randomly selected PCR products were examined by DNA sequencing, and the results were 100% concordant. 

### 2.3. Statistical Analysis

All data were analyzed using the SPSS software (version 19.0, SPSS Inc., Chicago, IL). To detect the agreement of the *IL-16* genotype frequency, Hardy-Weinberg equilibrium was tested with a goodness-of-fit *χ* ^2^ test. Demographic and clinical data between cases and controls were compared using the *χ* ^2^ test for dichotomous covariates and unpaired Student's *t*-test for continuous covariates. A binary logistic regression analysis was used to compute adjusted odds ratio (OR) and 95% confidence intervals (CI) for evaluating the association of the* IL-16* polymorphisms with IS. The criterion of statistical significance was set at *P* < 0.05.

## 3. Results 

The characteristics of cases and controls are summarized in Table 1. The genotype and allele frequencies of the rs4778889 T/C, rs11556218 T/G, and rs4072111 C/T polymorphisms in *IL-16 *gene are summarized in Table 2. The genotype distributions of the three polymorphisms in controls were consistent with the Hardy-Weinberg equilibrium. The genotype frequencies of the rs11556218 T/G polymorphism were significantly different between cases and controls. The TG genotype of the rs11556218 T/G polymorphism was associated with a significantly increased risk of IS compared with the TT genotype (adjusted OR = 1.88; 95% CI, 1.15–3.07). Similarly, the G allele of the rs11556218 T/G polymorphism was associated with a significantly increased risk of IS compared with the T allele (adjusted OR = 1.54; 95% CI, 1.05–2.27). However, there were no significant differences in the genotype and allele frequencies of *IL-16* rs4778889 T/C and rs4072111 C/T polymorphisms between the two groups, even after stratification analyses by age, gender, and the presence or absence of hypertension, diabetes mellitus, hypercholesterolemia, and hypertriglyceridemia.

## 4. Discussion

 Clinical and experimental data have demonstrated that inflammation plays fundamental roles in the pathogenesis of IS [2–7]. An increased level of *IL-16* mRNA and protein was observed in patients with inflammatory or autoimmune diseases, and the high expression of *IL-16* mRNA correlated with increased numbers of CD4+ cells in acute skin lesions [36, 37]. Ischemic stroke patients had a high risk for systemic infections, and anti-inflammatory agents may be a promising approach to treating IS [6, 38, 39]. 

 Based on the key roles of *IL-16* in inflammatory disorders, the association between *IL-16* polymorphism and the risk of inflammatory diseases has attracted widespread attention over the past decades. Some authors reported that the rs4778889 polymorphism was associated with increased risks of developing allergic contact dermatitis [25], Graves' disease [28], SLE [29], and endometriosis [26]. In contrast, some researchers reported that the rs4778889 polymorphism was not related to the risk of periodontitis [40] and CAD [30, 31]. In this study, we failed to find any association between the rs4778889 polymorphism and the risk of IS. These findings suggest that the rs4778889 polymorphism may have different roles in different diseases.

 Recently, another two nonsynonymous polymorphisms were identified (rs11556218 T/G and rs4072111 C/T). The two polymorphisms were reported to be associated with increased risks of developing SLE [29] and CAD [30, 31]. In conformity with the results, we found that the frequency of the rs11556218TG genotype in IS group was significantly higher than that in the control group, indicating that the *IL-16* rs11556218 polymorphism may be used as a genetic marker for IS.

 Although the mechanism of IL-16 as an inflammatory mediator is still not fully known, there is evidence that IL-16 participates in the inflammatory disorder through the activation of T lymphocytes and the secretion of inflammatory cytokines [41, 42]. In the early stages following an ischemic stroke, T lymphocytes were activated, released reactive oxygen species, and finally contributed to brain injury. In the later stages, T lymphocytes can regulate the repair and regeneration of the brain [41]. Depletion of T lymphocytes can reduce stroke size during the acute phase of ischemia, and the protective effect against stroke lasted in the later stages of infarct development [42]. This evidence may explain the possible role of *IL-16* gene polymorphism in IS. 

In conclusion, this study provides the first evidence for associations of *IL-16* gene polymorphisms with risk of IS in the Chinese population. The TG genotype and G allele of the rs11556218 polymorphism may have a promoting effect on IS. However, further studies are warranted in order to shed more light on the molecular mechanism.

## Figures and Tables

**Table 1:** Characteristics of study subjects.

Variables	IS (*n* = 198)	Controls (*n* = 236)	*P* value
Age (years, mean ± SD)	57.2 ± 11.8	55.4 ± 11.7	0.12
Gender (%)			
Male	122 (61.6)	155 (65.7)	0.38
Female	76 (38.4)	81 (34.3)	
Hypertension (%)	119 (60.1)	22 (9.3)	<0.001
Diabetes mellitus (%)	40 (20.2)	8 (3.4)	<0.001
Hypercholesterolemia (%)	27 (13.6)	26 (11.0)	0.41
Hypertriglyceridemia (%)	56 (28.3)	61 (25.8)	0.57

IS: ischemia stroke; SD: standard deviation.

**Table 2:** Genotype and allele distributions of the *IL-16* gene polymorphisms in IS cases and controls.

	IS cases *n* (%)	Controls *n* (%)	Adjusted OR (95% CI)	*P* value
rs4778889 T/C				
Genotypes				
TT	124 (62.6)	138 (58.5)	1.00	
TC	66 (33.3)	88 (37.3)	0.56 (0.16–1.98)	0.36
CC	8 (4.0)	10 (4.2)	0.94 (0.57–1.56)	0.82
Alleles				
T	314 (79.3)	364 (77.1)	1.00	
C	82 (20.7)	108 (22.9)	0.93 (0.61–1.40)	0.72

rs11556218 T/G				
Genotypes				
TT	88 (44.4)	136 (57.6)	1.00	
TG	102 (51.5)	94 (39.8)	1.88 (1.15–3.07)	0.01
GG	8 (4.0)	6 (2.5)	2.38 (0.63–9.05)	0.20
Alleles				
T	278 (70.2)	366 (77.5)	1.00	
G	118 (29.8)	106 (22.5)	1.54 (1.05–2.27)	0.03

rs4072111 C/T				
Genotypes				
CC	125 (63.1)	141 (59.7)	1.00	
CT	63 (31.8)	85 (36.0)	0.62 (0.37–1.04)	0.07
TT	10 (5.1)	10 (4.2)	1.38 (0.48–3.97)	0.55
Alleles				
C	313 (79.0)	367 (77.8)	1.00	
T	83 (21.0)	105 (22.2)	0.76 (0.49–1.16)	0.20

OR was adjusted for age, gender, hypertension, diabetes mellitus, hypercholesterolemia, and hypertriglyceridemia by binary logistic regression analysis.
